# Family resources, resilience beliefs, and parental adaptation: A moderated mediation analysis

**DOI:** 10.1111/famp.13067

**Published:** 2024-10-13

**Authors:** Anis Ben Brik, Yunqi Wang

**Affiliations:** ^1^ College of Public Policy Hamad Bin Khalifa University Doha Qatar; ^2^ Department of Child, Youth & Family Studies University of Nebraska–Lincoln Lincoln Nebraska USA

**Keywords:** ABC‐X model, family relationships, marital satisfaction, parental stress, resilience beliefs

## Abstract

Despite the extensive body of evidence documenting how pandemic‐related stressors (e.g., disruptions in daily routine) impact individuals' mental health, research examining family mechanisms through which stressors impact parental stress remains insufficient. The present study aims to address this gap by exploring a moderated mediation model that predicts parental stress resulting from the accumulation of pandemic‐related stressors. Specifically, we hypothesized a second‐stage moderated mediation model in which family resilience beliefs moderated the second‐stage indirect paths through family relationships and marital satisfaction, resulting in conditional indirect effects. Study data were collected from American parents (*n* = 1386). There was no evidence that family relationships and marital satisfaction mediated the association between stressor pile‐up and parent stress. In addition, family resilience beliefs did not significantly impact how marital satisfaction or family relationships affect parental stress. However, marital satisfaction mediated the relationship between stressor pile‐up and parental stress across all levels of family resilience beliefs. The findings of this study carry significant implications for post‐pandemic family interventions, suggesting the incorporation of resilience belief training and stress management strategies to improve intrafamilial communication.

The COVID‐19 pandemic has exerted a profound influence on mental well‐being, manifesting both directly, as a consequence of the illness and associated mortality, and indirectly, due to the psychological ramifications of public health interventions (Bae & Chang, 2023; Ben Brik et al., 2022). The repercussions of the pandemic have been acutely felt within the family as parents and offspring contend with an array of stressors, including isolation, disruption of routines, and an atmosphere of uncertainty regarding health and educational decisions (Gayatri & Irawaty, 2022; Tong et al., 2023). The confluence of these factors has engendered a significant exacerbation of psychological distress within families (Schmeer et al., 2021). Families encountered a variety of unparalleled strains, including disrupted structure and routines, intensified health anxieties, and economic adversity (Gruber et al., 2020). The closure of schools and childcare facilities and the shift to remote work significantly impacted family routines (McGoron et al., 2022). The threat of COVID‐19 infection has amplified health‐related worries for parents as they navigate to keep their families safe (Johnson et al., 2021). Additionally, parents experienced stress related to managing their children's mental health, with increased anxiety and depression observed in children and adolescents during the pandemic (Calvano et al., 2023). Managing their mental health and children's needs contributed to elevated parental stress (Giordano et al., 2022). Moreover, economic adversity caused by the pandemic, such as job loss or reduced income, has significantly impacted parental stress (Reich et al., 2023).

Despite the extensive body of evidence documenting the adverse effects of pandemic‐related stressors on mental health, there remains a dearth of research examining the family mechanisms through which these stressors impact parental stress. Family mechanisms refer to the processes and dynamics occurring within families that have the potential to influence individual well‐being, whether positively or negatively. Such mechanisms include family relationships, communication, support, and personal and familial coping strategies. Previous findings documented how families adapted positively to the rapid changes in the face of the pandemic (Gayatri & Irawaty, 2022; Prime et al., 2022). Some of them reported having increased time spent together with their family members and involving in the shared problem‐solving process (Prime et al., 2022). However, the examination of roles of family resources (e.g., relational support) and functioning (e.g., family positive beliefs about resilience) have still not been fully discussed. The present study aims to address this gap by exploring a moderated mediation model that predicts parental stress resulting from the accumulation of pandemic‐related stressors.

## Conceptual framework

**FIGURE 1:**
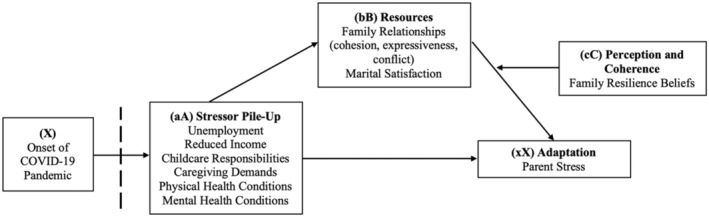
Conceptual model.

This study is guided by the Double ABC‐X model of family stress and adaptation (McCubbin & Patterson, 1983). This model conceptualizes the family unit as a system striving to maintain equilibrium for effectively addressing members' developmental, instrumental, and material needs. The Double ABC‐X model (Figure 1) explores the interplay between the stressful event or crisis (A), family's resources (B), the family's perception and appraisal of the event (C), and their adaptation or response to the event (X; McCubbin & Patterson, 1983). In addition, the Double ABC‐X model encompasses predictor variables: (aA) stressor pile‐up, (bB) existing adaptive resources, and (cC) family members' perceptions and coherence of the stressor (Rosino, 2016). The outcome variable (xX) denotes the degree to which the stressor triggers a new crisis, jeopardizing the family's functioning and well‐being.

In the context of the pandemic, (aA) encapsulates the direct consequences of the pandemic, reflecting significant changes in families' social contexts and norms, such as increased caretaking and childcare demands and job‐related challenges leading to income loss. Pre‐existing physical and mental health challenges in family members may also be exacerbated due to the pandemic and contribute to stressor pile‐up. Variable (bB) captures the adaptive resources available to the family, which can influence the impact of stress on family outcomes (Boss, 2002). Variable (cC) refers to a family's perceptions and understanding of the stressful situation under the COVID‐19 pandemic, including evaluations of stressors and their impacts. These beliefs could function independently from existing adaptive resources that families have. For instance, a family may have adequate existing resources, yet family members hold negative beliefs about their resilience in the face of adversity, which could affect parental adaptations eventually. Family resilience beliefs may help families cope with the mental health effects of the pandemic. These beliefs affect mental health in stressful life circumstances by influencing individuals' interpretation of adverse events and fostering a positive outlook despite challenges (Walsh, 1996). While the original Double ABC‐X model (McCubbin & Patterson, 1983) did not specify the directionality between stressors and existing resources, the current study proposes a new perspective. It suggests that intensified pandemic‐related stressors may affect adaptations (xX factors), with existing resources (bB) potentially mediating this relationship by influencing how stress impacts family outcomes (i.e., adaptation). This proposal considers that stressor pile‐up was exacerbated by the context of the COVID‐19 pandemic as a global health crisis (Condon et al., 2024).

### Stressor pile‐up during the pandemic

Amid the pandemic, a myriad of stressors has emerged, encompassing economic stressors (e.g., loss of income), health‐related stressors (e.g., fears related to infection), lockdown‐related stressors (e.g., loss of child care), and grief‐related stressors (e.g., loss of loved ones; Lotzin et al., 2022). Economic stressors, including job loss and diminished income, have adversely impacted family well‐being (Lengua et al., 2022). Health‐related stressors entail challenges in procuring medical support services and the grief ensuing from losing loved ones (Gruber et al., 2021). The cumulative effects of these stressors have been consistently associated with detrimental consequences on individuals' mental health and overall well‐being (Graupensperger et al., 2022). Previous research indicates that parents who have encountered multiple stressors during the pandemic have been disproportionately affected by the mental health sequelae of COVID‐19 (Lewis et al., 2022). This underscores the profound influence of stressor pile‐up on mental health and family functioning in the context of the pandemic.

### Family relationships and parent stress

Family relationships are crucial to family functioning, marital satisfaction, and parental mental health, encompassing nuclear and extended family connections (Gillespie et al., 2014). Positive relationships contribute to increased satisfaction in family life and improved mental health outcomes (Amato & Keith, 1991; Gillespie et al., 2014). Effective communication and conflict resolution are essential for cultivating positive family relationships (Gottman, 1997). During the pandemic, strong family relationships, characterized by support and cohesion, have been associated with reduced stress levels among parents (Feinberg et al., 2021). Previous studies have shown that family support is significantly associated with diminished stress levels among parents during the pandemic (Li & Xu, 2020).

### Marital satisfaction, resilience beliefs, and parental stress

Marital satisfaction, encompassing how individuals experience contentment and fulfillment within their romantic partnerships, can influence parental stress, a phenomenon characterized by the strain and pressure parents endure in addressing their familial obligations (James et al., 2022). Furthermore, marital satisfaction may suffer adverse effects from pandemic stressors, potentially yielding considerable implications for the psychological well‐being of family members. Consistently, extant research has corroborated the existence of an inverse relationship between stress and marital satisfaction, particularly concerning external stressors (Randall & Bodenmann, 2017). Such external stressors, including financial adversities and health‐related issues, have been documented to exacerbate pre‐existing relational challenges and engender new difficulties during other natural disasters (Cohan, 2010). Given the heightened stress levels that numerous families confronted amidst the pandemic, an amplified risk of discord within romantic relationships ensued (Schmid et al., 2020).

### Family relationships, marital satisfaction, and parental stress

The interplay between family relationships and marital satisfaction may elucidate the association between stressor accumulation and parental stress. Stressor pile‐up pertains to the convergence of numerous stressors, resulting in heightened stress levels and tension (McCubbin & Patterson, 1983). Positive family relationships are associated with increased family satisfaction, diminished stress and conflict, and improved mental health outcomes for all family members (Amato & Keith, 1991; Olson & DeFrain, 2000). Positive family relationships may also attenuate the repercussions of stressor pile‐up on parental stress by engendering a supportive milieu that cultivates resilience and efficacious coping mechanisms (Walsh, 2016). In addition, marital satisfaction may serve an explanatory function in the nexus between stressor pile‐up and parental stress. Studies have indicated that marital satisfaction may be detrimentally affected by external stressors, encompassing stressor pile‐up, which subsequently influences parental stress (Randall & Bodenmann, 2017). High levels of marital satisfaction have been associated with increased family resilience, cohesion, and communication (Cuzzocrea et al., 2013). In contrast, diminished marital satisfaction may intensify the impact of stressor pile‐up on parental stress, as it may precipitate escalated conflict and reduced emotional support within the familial context (Bodenmann, 2005).

## The present study

The overarching objective of the current study is to explore parents' experience of stress during the pandemic within the ABC‐X conceptual framework. We conceptualize family relationships and marital satisfaction as adaptive resources or factors that influence the relationship between parents' experience of pandemic‐related stressors and their experience of stress. Parent resilience beliefs were included in the model as C factors (i.e., evaluation of stressors and their likely impacts) that may interact with marital satisfaction to determine parents' stress (Rosino, 2016) jointly. In this study, we test these premises using a moderated mediation analytic approach to examine the role of parent resilience beliefs and marital satisfaction in jointly determining parental stress levels. This approach addresses questions regarding stressor pile‐up, family relationships, and resilience beliefs and seeks to capture the complexities and contingencies of stress‐related mechanisms. Ultimately, the study contributes to a better understanding of how parents can cope with stressors during challenging times and fosters positive growth within families. Specifically, the research questions explore whether the relationship between stressor pile‐up (aA) and adaptation (xX) is mediated by adaptive resources (bB) and whether the proposed mediated relationships are influenced by perceptions and coherence (cC). Figure 1 depicts a graphical representation of our hypotheses. Stressor pile‐up serves as the focal predictor, consisting of pandemic‐related stressors such as unemployment, loss of income, childcare difficulty, and caregiving challenges; while family relationships (cohesion, expressiveness, and conflict) and marital satisfaction function as mediators. In addition, family resilience beliefs were considered as the moderator, while parent stress symptoms served as the dependent variable. Covariates included age, gender, race, educational attainment, and employment status. We hypothesized a second‐stage moderated mediation model, in which family resilience beliefs moderated the second‐stage indirect paths through family relationships and marital satisfaction, resulting in conditional indirect effects.

## METHOD

### Participants and data collection

Data for the current study were drawn from the *COVID‐19 Family Life Study* (Ben Brik, 2020), which sought to investigate the impact of the COVID‐19 pandemic on families across cultures. The original dataset was collected from 72 countries across all inhabited continents, representing over 75% of the global population. The current study focuses solely on the U.S. sample collected through an online panel from a research company. This sample comprises 2026 participants, representing age, sex, race‐ethnicity, education, and marital status nationally. Prospective participants were contacted via email and invited to participate. Individuals who provided informed consent by clicking a box on the online survey introduction page were then presented with the survey questions. Data collection occurred between September and October 2020, following institutional review boards' approval—Hamas Bin Khalifa University, QBRI‐IRB 2020‐06‐021—of the study design and materials. The analytic sample for the present study comprises a subset of 1386 participants who reported being married or cohabiting with a romantic partner. Participants were chosen from an online panel to ensure national representativeness regarding age, sex, race‐ethnicity, education, and marital status through a quota sample of American parents with children under 18. There was one respondent (participant) per family in the study.

Regarding the relationship with the children living in the household, 87% of participants identified themselves as biological parents, 4.5% as step‐parents, 2.9% as grandparents, 1.0% as being in this role due to COVID‐19, and 2.7% reported other relationships. Among the participants who identified themselves as biological parents, 56.7% were fathers, and 43.3% were mothers. Table 1 presents an in‐depth overview of the analytic sample's socio‐demographic characteristics.

**TABLE 1:** Demographic characteristics of the sample (*N* = 1386).

Variables	Mean	SD
Age, years	36.7	9.3
	*n*	%
Gender		
Male	782	56.4
Female	604	43.6
Educational attainment		
Less than high school	28	2.0
High school diploma	294	21.2
Some college	344	24.8
College degree	278	20.1
Post‐graduate	442	31.9
Race/ethnicity, *n* (%)		
White	970	70.0
Black or African American	124	8.9
Hispanic	196	14.1
American Indian or Alaska Native	22	1.6
Asian	60	4.3
Other	14	1.0
Employment status		
Student, not working	32	2.3
Self‐employed	62	4.5
Part‐time employment	118	8.5
Full‐time employment	914	65.9
Unable to work due to disability	40	2.9
Homemaker/stay at home parent	164	11.8
Unemployed and seeking work	42	3.0
Retired	14	1.0

### Measurement

#### Stressor pile‐up (aA factor)

Consistent with methodologies employed in prior research on the ABC‐X model (Ben Brik et al., 2024), a composite variable indicative of stressor accumulation was constructed by aggregating dichotomous variables. The stressor pile‐up scale was computed from the summation of the following variables: (a) employment loss attributed to the COVID‐19 pandemic (no = 0, yes = 1), (b) decreased income due to the COVID‐19 pandemic (no = 0, yes = 1), (c) adjusted work schedules in response to shifting childcare obligations during the COVID‐19 pandemic (no = 0, yes = 1), (d) providing care for a child with unique educational requirements (no = 0, yes = 1), (e) assuming caregiving duties for an older relative or acquaintance (no = 0, yes = 1), (f) offering care for an individual with a chronic health issue or disability (no = 0, yes = 1), (g) the existence of one or more chronic physical health disorders in oneself or another household occupant (no = 0, yes = 1), and (h) the existence of one or more chronic mental health disorders in oneself or another household occupant (no = 0, yes = 1). The resulting composite variable exhibited a potential range of 0–8. The stressor pile‐up scale displayed robust internal reliability in the current study, as evidenced by a Cronbach's α coefficient of 0.92. Moreover, Confirmatory Factor Analysis (CFA) was conducted using AMOS (Version 26) with full information maximum likelihood to examine the goodness‐of‐fit of stressors. The model had a good fit as χ2/df = 10.09, CFI = 0.92, TLI = 0.91, RMSEA = 0.07 [0.12–0.15], and SRMR = 0.05 (Hu & Bentler, 1998).

#### Family relationship (bB factor)

The Family Environment Scale (FES; Moos & Moos, 1981) was employed in this study. This 90‐item self‐report instrument evaluates family functioning across ten domains, encompassing interpersonal connections, personal development, and system maintenance. The FES is a widely recognized tool in family therapy and research, demonstrating reliability and validity in measuring the family environment. It is frequently used to examine the influence of family dynamics on individual and family functioning and to inform the creation of interventions that enhance family functioning. In the present study, we focused on the aspect of family dynamics, which comprised three nine‐item sub‐scales: Cohesion (e.g., “*Family members genuinely support and inspire one another*”), Expressiveness (e.g., “*We share our concerns*”), and Conflict (e.g., “*Our family experiences frequent disagreements*”). Parents were prompted to rate items on a Likert scale ranging from 1 (*strongly disagree*) to 7 (*strongly agree*). Higher scores denote a more favorable impact on the family environment. In our sample, the reliabilities for the cohesion (*α* = 0.88), expressiveness (*α* = 0.94), and conflict (*α* = 0.92) sub‐scales were deemed acceptable, consistent with prior studies (Margalit & Heiman, 1986).

#### Marital satisfaction (bB factor)

The bB factor was assessed using the ENRICH Marital Satisfaction Scale (EMS; Fowers & Olson, 1993). This scale serves as a concise measure of romantic relationship quality (Fowers & Olson, 1993). Participants indicate their level of agreement with 15 statements using a 5‐point Likert scale, where 1 = Strongly disagree, 2 = Disagree, 3 = Neither agree nor disagree, 4 = Agree, and 5 = Strongly agree. Sample items include “*My partner and I have a complete understanding of each other*,” “*I am highly satisfied with our decision‐making and conflict resolution process*,” and “*I am extremely pleased with how we manage our leisure activities and the time we spend together*.” After reverse scoring several items so that higher scores represent more positive evaluations, items are summed to compute raw scores for two scales: Marital Satisfaction (10 items) and Idealistic Distortion (5 items). Raw scale scores are converted to percentile scores using national norms established for this questionnaire. An overall individual EMS score is calculated by correcting the Marital Satisfaction percentile score downward based on the extent to which the respondent portrays the marriage in an unrealistically optimistic manner, using the following formula where PCT = percentile score for the Marital Satisfaction scale and ID = percentile score for the Idealistic Distortion scale: EMS score = PCT‐[(0.40 × PCT) (ID × 0.01)]. The internal reliability of the EMS used in the current study yielded a Cronbach's alpha of 0.94. Moreover, prior research has supported the scale's criterion and construct validity (Fowers & Olson, 1993).

#### Family resilience beliefs (cC factor)

The cC factor was assessed using ten items selected from the original 25‐item Connor‐Davidson Resilience Scale (CD‐RISC‐10). The instrument assesses resilience, encompassing aspects of adaptability and stress‐management capabilities. Sample items include “*I tend to recover after illness or adversity*,” “*I persevere even when the situation appears hopeless*,” and “*dealing with stress strengthens me*.” A 5‐point Likert scale was employed (0 = *not true at all*, 4 = *true all the time*). A respondent's total score can range from 0 to 40, with higher scores signifying greater resilience levels. This concise, unidimensional version maintains excellent psychometric properties, comparable to the more extended version, which has been deemed suitable for use across diverse cultures and widely applied in epidemiological studies (Connor & Davidson, 2003). In this study, the alpha coefficient for internal consistency was 0.92, aligning with previous research on the original English version (Connor & Davidson, 2003).

#### Parent stress symptoms (xX factors)

The xX factors were assessed using the Depression, Anxiety, and Stress Scale‐21 Items (DASS‐21; Norton, 2007). The DASS‐21 is a globally recognized instrument for assessing symptoms of depression, anxiety, and stress (Scholten et al., 2017). The DASS‐21 has been validated in clinical samples (Antony et al., 1998; Lovibond & Lovibond, 1995). Following Norton (2007), we employed the full 21‐item scale with a response range of 0 (*did not apply to me at all*) to 3 (*applied to me very much, or most of the time*) rather than dividing the scale into its separate components. Example items include, “*I found it hard to wind down*,” “*I felt depressed and had no motivation*,” and “*I felt I had no desire for anything*.” Responses were totaled across items to produce an aggregate score. DASS‐21 scores are determined by summing the relevant items' scores per scale. Then, the DASS‐21 subscale total is multiplied by two to yield the final score for categorization into average (0–9 for depression, 0–7 for anxiety, and 0–14 for stress), mild/moderate (10–20 for depression, 8–14 for anxiety, and 15–25 for stress), or severe/extremely severe (21+ for depression, 15+ for anxiety, and 26+ for stress; Lovibond & Lovibond, 1995). In the current sample, the DASS‐21 scores had Cronbach's alphas of 0.87, consistent with previous research on the original English version (Lovibond & Lovibond, 1995).

### Covariates

Sociodemographic variables, such as parental age, gender, ethnicity, educational achievement, and employment status, were incorporated as covariates. Education was classified into five categories (less than high school, high school diploma, some college, college degree, and postgraduate). Employment status was divided into seven groups (self‐employed, part‐time, full‐time, unable to work due to disability, homemaker/stay‐at‐home parent, unemployed and looking for work, and retired).

### Statistical approach

IBM SPSS Statistics (Version 28.0) was utilized to perform all statistical analyses. Preliminary analyses included screening data for outliers and ensuring continuous variables conform to a normal distribution (Tabachnick & Fidell, 2013). Skewness, kurtosis values, and normality test results (e.g., Shapiro–Wilk test) were examined to confirm that continuous study variables followed a normal distribution. All values fell within acceptable ranges to infer normality. Given missing values were below 5% of the total number of values, we thereby replaced the missing data using the multiple imputation by chained equations (MICE) in R package (Van Buuren & Groothuis‐Oudshoorn, 2011). The hypothesized moderated mediation model (Figure 1) was tested in a singular model using a bootstrapping method to determine the significance of the indirect effects at various levels of the moderator (Hayes, 2013). The predictor variable was stressor pile‐up, with family and relationship satisfaction as mediators. Including family satisfaction and relationship satisfaction in the mediation model concurrently (rather than estimating separate models for each variable) provides estimates of the indirect and direct effects unique to each variable, addressing potential correlation concerns between the mediator (Hayes & Rockwood, 2017). The outcome variable was parental stress symptoms, and the suggested moderator was family resilience beliefs. Moderated mediation analyses examine the conditional indirect effect of a moderating variable (i.e., family resilience beliefs) on the association between a predictor (i.e., stressor pile‐up) and an outcome variable (i.e., stress) via potential mediators (i.e., family satisfaction and relationship satisfaction).

The PROCESS macro, model 14, v4 (Hayes, 2013) with bias‐corrected 95% confidence intervals, was employed to test the significance of the indirect (i.e., mediated) effects moderated by family resilience beliefs, conditional indirect effects. This macro's advantage is that it implements recommended bootstrapping procedures and automatically calculates post hoc probing for moderating effects. The model was estimated using 5000 bootstrapped samples. To assess moderated mediation, the significance of the conditional indirect effect was estimated at the moderators' 16th, 50th, and 84th percentile values. Confirmation of moderated mediation was based on the index of moderated mediation (Hayes, 2015). Similar to traditional moderation analyses where a significant interaction indicates that the simple slopes differ from one another (Aiken & West, 1991), a significant index of moderated mediation suggests that the moderator is linearly related to the indirect effect and implies that the conditional indirect effects defined by the two distinct values of the moderator are statistically different. The significance of the index of moderated mediation (i.e., evidence of moderation of the indirect effects of the relations between stressor pile‐up and parental stress by family satisfaction and relationship satisfaction) is established when the bootstrap confidence interval for the index of moderated mediation does not include zero.

## RESULTS

### Correlation analysis

Descriptive statistics and bivariate correlations among the variables examined in this study are summarized in Table 2. Stressor pile‐up was negatively associated with family resilience beliefs (*r* = −0.192, *p* < 0.001), parent stress (*r* = −0.280, *p* < 0.001), and positively associated with family relationship (*r* = 0.055, *p* < 0.01). Higher family relationship was associated with high marital satisfaction and high parent stress (*r* = 0.163 and 0.161, *p* < 0.001), and lower family resilience beliefs (*r* = −0.211, *p* < 0.001). Moreover, high marital satisfaction was associated with lower family resilience beliefs and lower parent stress (*r* = −0.281 and −0.245, *p* < 0.001).

**TABLE 2:** Descriptive statistics and correlations among variables (*N* = 1386).

Variables	Mean	*SD*	Range	1	2	3	4
Stressor Pile‐up	2.68	1.61	0–8	_			
2 Family Relationships	58.98	8.10	36–84	−0.047	_		
3 Marital Satisfaction	47.01	11.64	9–81	0.055*	0.163**	_	
4 Family Resilience Beliefs	29.49	8.48	0–40	−0.192**	−0.211**	−0.281**	_
5 Parent Stress	8.86	6.18	0–21	−0.280**	0.161**	−0.245**	0.075**

* *p* < 0.01.

** *p* < 0.001.

### Mediation analyses

After adjusting for factors including gender, age, race, educational attainment, and employment status, mediation analysis (Table 3) indicated that an association between stressor pile‐up and both family relationships (*B* = −0.0022, *SE* = 0.0012, *p* = 0.0724) and marital satisfaction (*B* = 0.0480, *SE* = 0.0253, *p* = 0.0579) was statistically non‐significant. Moreover, stressor pile‐up (*B* = −0.3477, *SE* = 0.0350, *p* = 0.000) was negatively associated with parental stress. Likewise, family relationship (*B* = 4.8322, *SE* = 0.7804, *p* = 0.0000) and marital satisfaction (*B* = −0.3788, *SE* = 0.0379, *p* = 0.0000) were associated with parent stress. These findings suggest that stressor pile‐up is negatively associated with parental stress but not significantly associated with family relationships or marital satisfaction. On the other hand, the findings also show that family relationships and marital satisfaction are significantly associated with parent stress. Specifically, a positive family relationship and high marital satisfaction are associated with lower parent stress levels. This suggests that having a supportive and positive family relationship and a high‐quality relationship with a partner can help reduce stress in parents. In other words, the negative association between stressor pile‐up and parent stress may be partially explained by the positive association between family relationships and parent stress and the negative association between marital satisfaction and parent stress.

**TABLE 3:** Regression results for hypothesized model of moderated mediation.

Antecedent	Consequent								
	M_1_: Family relationships			M_2_: Marital satisfaction			Y: Parent stress		
	*B*	*S*.E.	*p*	*B*	*S*.E.	*p*	*B*	*SE*	*p*
Stressor Pile‐up	−0.0022	0.0012	0.0724	0.0480	0.0253	0.0579	−0.3477	0.0350	0.0000
Family Relationships	–	–	–	–	–	–	4.8322	0.7804	0.0000
Marital Satisfaction	–	–	–	–	–	–	−0.3788	0.0379	0.0000
Family Resilience Beliefs (RES)	–	–	–	–	–	–	0.0184	0.0344	0.5938
Family Relationships x RES	–	–	–	–	–	–	−0.1865	0.0468	0.0001
Marital satisfaction x RES	–	–	–	–	–	–	0.0030	0.0025	0.2433
	*R* ^*2*^ = 0.0394			*R* ^*2*^ = 0.0270			*R* ^*2*^ = 0.2198		
	*F* (6,1379) = 9.4185**			*F* (6,1379) = 6.3852**			*F* (11,1374) = 35.1839**		

	Conditional indirect effects at three levels of the moderator						
	Effect	S.E.	L.L. 95% CI	UL 95% CI	Index of Moderated Mediation		
Family Relationships					0.0004, CI [0.0000, 0.0010]		
Low RES	−0.0163	0.0097	−0.0369	0.0016			
Medium	−0.0104	0.0062	−0.0237	0.0010			
High RES	−0.0046	0.0036	−0.0130	0.0006			
Marital Satisfaction					0.0001 [−0.0001, 0.0006]		
Low RES	−0.0200	0.0109	−0.0438	−0.0006			
Medium RES	−0.0179	0.0093	−0.0367	−0.0006			
High RES	−0.0159	0.0082	−0.0329	−0.0005			

*Note*: Unstandardized regression coefficients reported. Age, gender, race, employment status, and educational attainment included as covariates. Bootstrap sample = 5000.

Abbreviations: CI, confidence interval; L.L., lower limit; U.L., upper limit.

**p < 0.001.

### Moderated mediation analyses

**FIGURE 2:**
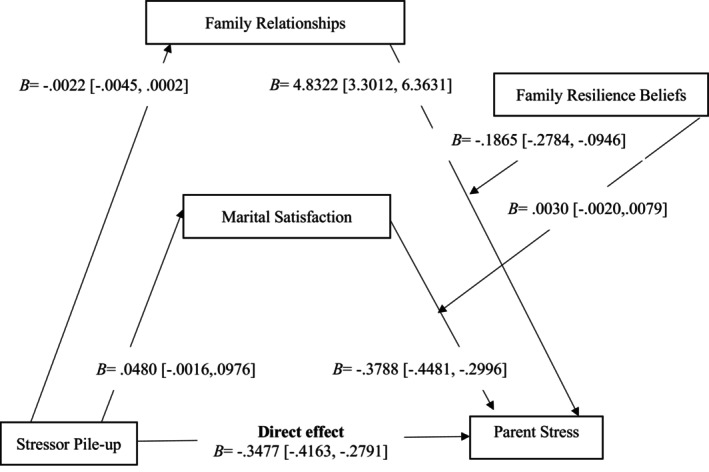
Statistical model.

**FIGURE 3:**
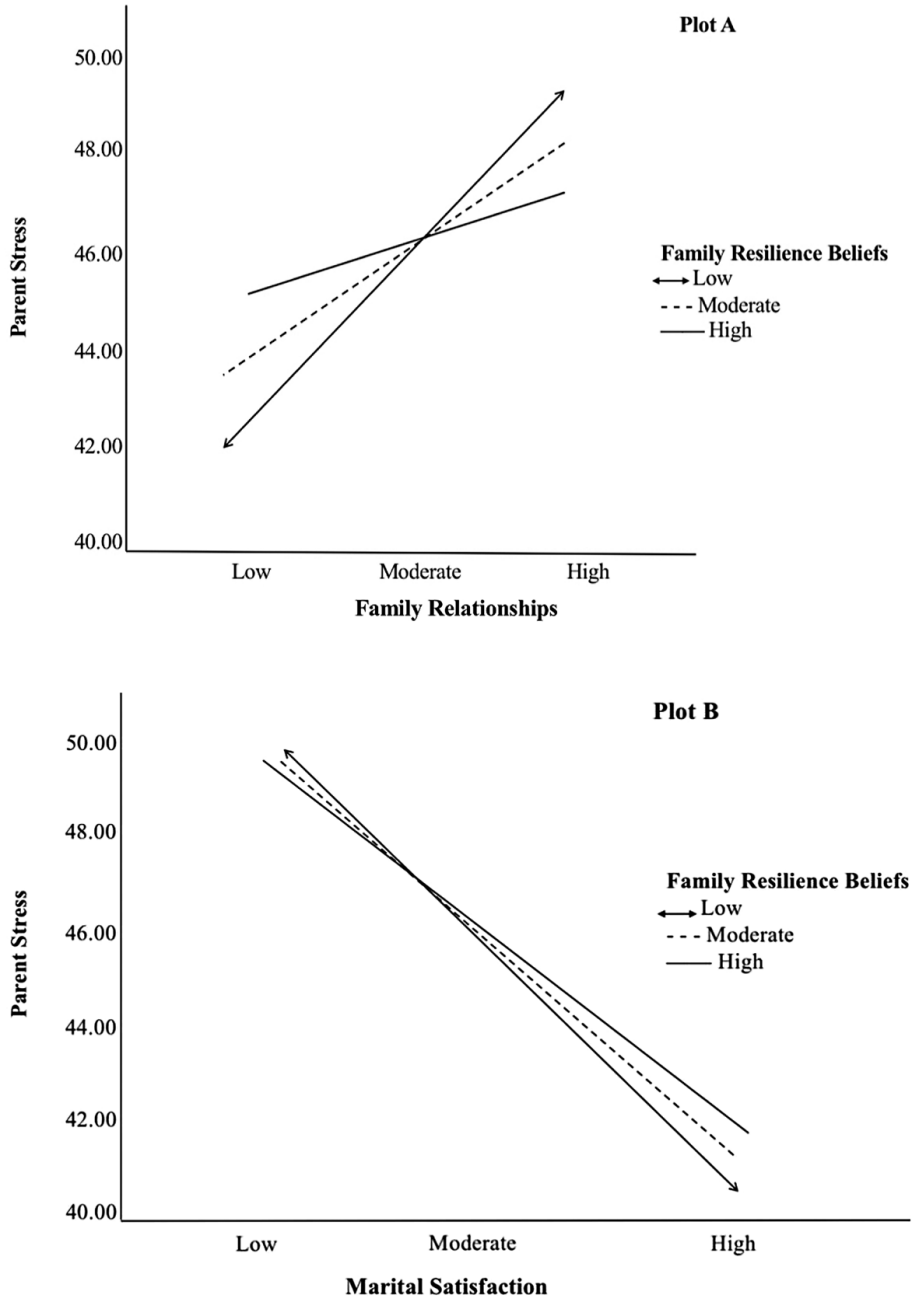
Conditional direct effects of family relationships and marital satisfaction on stress on levels of family resilience beliefs.

The hypothesized moderated mediation model was tested using the PROCESS macro model number 14, which tests a model whereby family resilience beliefs moderate the relations of family satisfaction and marital satisfaction to parent stress (Figure 1; Hayes, 2013). Results of this analysis are presented in Table 3 and depicted visually in Figure 2. The direct association between family relationships and parent stress was moderated by family resilience beliefs (*B* = −0.1865, *SE* = 0.0468, *p* = 0.0001). These findings suggest that family resilience beliefs moderate the association between family relationships and parent stress. In other words, the strength or direction of the relationship between these two variables may be influenced by an individual's beliefs about their family's resilience. However, family resilience beliefs did not moderate the direct association between marital satisfaction and parent stress (*B* = 0.0030, *SE* = 0.0025, *p* = 0.2433). Figure 3 visually depicts the interactions between family relationships and family resilience beliefs (plot A) and marital satisfaction and family resilience beliefs (plot B). Plot A was constructed by estimating the simple effect of family relationships on stress scores for low, moderate, and high values of family resilience beliefs.

Similarly, plot B was constructed by estimating the simple effect of marital satisfaction on stress scores for the three levels of family resilience beliefs. As shown in Figure 3 Plot A, the family relationship was positively associated with stress for parents with low, moderate, and high family resilience beliefs. As family relationships increased from Low to High, the effect on parent stress varied depending on the level of family resilience beliefs. For families with low resilience beliefs, parent stress increased sharply as family relationships increased. For families with moderate resilience beliefs, parent stress increased more gradually. For families with high resilience beliefs, parent stress increased only slightly as family relationships increased. As depicted by the steepness of the slopes, the positive relationship between family relationships and parent stress was strongest among individuals with low family resilience beliefs, moderate for those with moderate resilience beliefs, and weakest for those with high resilience beliefs.

Figure 3 Plot B illustrates that family resilience beliefs moderated the association between marital satisfaction and parent stress. Here, results revealed that marital satisfaction was negatively associated with parent stress at low, moderate, and high levels of family resilience, with the strongest association for parents with low family resilience beliefs. A formal test of moderated mediation based on the index term revealed that there was no evidence that family relationships (Index = 0.0004, 95% CI = 0.0000, 0.0010) and marital satisfaction (Index = 0.0001, 95% CI = −0.0001, 0.0006) mediated the association between stressor pile‐up and parent stress. Further hypothesis tests were conducted to determine whether the conditional indirect effect was statistically significant at values corresponding to low (18), moderate (27), and high (36) values of family resilience beliefs. PROCESS automatically generates these conditional indirect effects at moderator values corresponding to the sample data's 16th, 50th, and 84th percentile points. There was no evidence that family relationships mediated the association between stressor pile‐up and parent stress across levels of family resilience beliefs. Furthermore, marital satisfaction mediated the association between stressor pile‐up and stress across all levels of family resilience beliefs, as follows: low family resilience beliefs (effect = −0.0200, CI = −0.0438, −0.0006); moderate family resilience beliefs (effect = −0.0179, CI = −0.0367, −0.0006); and high family resilience beliefs (effect = −0.0159, CI = −0.0329, −0.0005). These findings indicate no evidence that family resilience beliefs moderate the second‐stage indirect paths of marital satisfaction or family relationships in a second‐stage moderating mediation model. The results of the formal test of moderated mediation and the hypothesis tests indicate no evidence that family relationships or marital satisfaction mediate the relationship between stressor pile‐up and parent stress. However, the results indicate that marital satisfaction mediates the relationship between stressor pile‐up and stress across all levels of family resilience beliefs. Family resilience beliefs moderate the effect of marital satisfaction on parent stress. The effect is more substantial at lower levels of family resilience beliefs and weaker at higher levels. The results do not uphold our hypothesis that family resilience beliefs would moderate the second‐stage indirect paths of marital satisfaction or family relationships in a second‐stage moderating mediation model.

## DISCUSSION

This study explores factors within the Double ABC‐X model of family stress and adaptation that could elucidate the stress experienced by parents during the pandemic and the implications for long‐term adaptation (McCubbin & McCubbin, 1988). We examined the moderating role of family resilience beliefs in the relationship between family functioning and parent stress and the mediating role of family relationships and marital satisfaction in the relationship between stressor pile‐up and parent stress. Our findings contribute to the literature delineating family mechanisms linking COVID‐19 stressors with stress symptoms in parents. Moreover, our application of a moderated mediation analytic approach extends prior work by explicitly testing the conditional indirect effects theorized in the ABC‐X conceptual framework among stressor pile‐up, adaptive resources, perception and coherence factors, and adaptive outcomes. The findings suggest that family resilience beliefs did not significantly impact how marital satisfaction or family relationships affect parental stress. The Family System Theory (Bowen, 1978) suggests an explanation of the findings, which posits that a family functions as a system, with each member's actions and behaviors affecting the entire family unit. In the context of this study, marital satisfaction and family relationships were more directly associated with parent stress than family resilience beliefs. The dynamics within the family system may substantially influence parent stress, making the role of family resilience beliefs less significant in this context. Consequently, family resilience beliefs might not significantly moderate marital satisfaction, family relationships, and parent stress. An alternative interpretation of the results is suggested by the Social Support Theory (Cohen & Wills, 1985), which suggests that social support from family members can buffer the adverse effects of stressors and promote resilience. In the case of this study, the level of social support provided by the spouse and other family members may have a more direct impact on parent stress than family resilience beliefs. As a result, the role of family resilience beliefs in moderating the relationship between marital satisfaction, family relationships, and parent stress might be less significant. Moreover, our findings indicate that marital satisfaction mediates the relationship between stressor pile‐up and stress across all levels of family resilience beliefs. An explanation is suggested by Dyadic Coping Theory (Bodenmann, 2005), which posits that the way couples cope with stress together can significantly impact their relationship quality and individual well‐being. In this study, marital satisfaction may mediate the relationship between stressor pile‐up and stress because effective dyadic coping can alleviate the adverse effects of accumulated stressors on both partners, regardless of their family resilience beliefs. These findings support the ABC‐X model of family adaptation (McCubbin & McCubbin, 1988), particularly the role of marital satisfaction in coping with stressors. However, the study did not find evidence that family relationships or marital satisfaction mediated the relationship between stressor pile‐up and parental stress, suggesting that the ABC‐X model may only capture some of the complexity of the relationships between its components. This highlights the need for continued testing and refinement of the model to understand family adaptation to stressors better (Conger et al., 1992). Nevertheless, the findings of the current study provide implications for programs and workshops in the post‐pandemic era, specifically, to implement resilience beliefs training and stress management strategies to enhance positive communication within families. This also applies to couples therapy, especially interventions that focus on enhancing marital satisfaction via conflict resolution skills. The clinical implications of the findings are significant for practitioners working with families experiencing stress. Enhancing marital satisfaction can protect against parental stress, which is crucial for effective family functioning. Interventions such as the Prevention and Relationship Enhancement Program (PREP) have effectively improved relationship quality and alleviated parental stress (Ben Brik et al., 2024). Moreover, family resilience beliefs are critical in moderating the relationship between family dynamics and parental stress. Clinicians should incorporate resilience‐building strategies into their therapeutic practices, using tools such as the Family Resilience Assessment Scale (FRAS) to identify specific areas for intervention (Sixbey, 2005). Workshops or therapy sessions that enhance communication and emotional support between partners while encouraging resilience strategies can empower families to navigate stressors effectively (Walsh, 2016). Additionally, our findings should be interpreted within the context of scholarship on resilience. Recent research has emphasized that resilience is not merely an individual or family trait but a dynamic process influenced by multiple ecological systems (Ungar, 2011). This perspective aligns with our findings on the complex interplay between stressors, family relationships, and resilience beliefs. Moreover, critical perspectives on resilience highlight the importance of considering cultural and contextual factors in resilience processes (Bottrell, 2009). While providing valuable insights, our study may need to fully capture how cultural contexts and socioeconomic factors influence family resilience beliefs.

### Limitations and future directions

The present study has several limitations that should be considered when interpreting the results. Firstly, the sample of participants in this study could have represented the full diversity of the U.S. population. Although the sample was large, it was comprised primarily of people who identified as White. The cultural diversity across and within the United States calls for increased representation of the entire population in future studies to improve the generalizability of findings to marginalized groups. Secondly, the cross‐sectional design of this study hinders the establishment of causal relationships among the variables. Given the potential bias that cross‐sectional design could introduce in the mediation models (Maxwell et al., 2011), results of the current study should be interpreted with caution. The mediating role of family existing resources (i.e., marital satisfaction) in the relationship between stressor pile‐up and parental stress, across different levels of resilience beliefs, may only apply to this study's sample during the first wave of the COVID‐19 pandemic. Further research in the post‐pandemic era is needed to capture the potentially devastating consequences of this global health crisis, as the current study only examined the first wave of the pandemic, while parental stress and family dynamics may shift over time. Additionally, because of other pandemic‐specific challenges (such as quarantine), the data collection during the initial phase of the pandemic was limited to online options. Considering this study adopted convenience sampling and the online‐based collection method, certain potential participants could be less likely to be included in the sample, such as individuals who do not have access to the internet during the pandemic. The inclusion of dyad variables such as length of marriage could be helpful to examine their effects on marital relationships in family dynamics. Future studies adopting hybrid data collection approaches (e.g., face‐to‐face interviews) could also better understand parental stress and adaptation and support families.

### Conclusion

This study examines the factors within the Double ABC‐X model that could clarify parental stress during the pandemic. This study contributes to a growing literature investigating stress, family relationships, and resilience during the COVID‐19 pandemic. A unique contribution of the present study is that we considered two aspects of family relational well‐being—one global and one more specific to an important family sub‐system. Our results highlight an explanatory role for family relationships and marital satisfaction in understanding the association between pandemic stressors and stress symptoms in parents. An important direction for future research is the potential impact of resiliency beliefs on stress for some parents. The results have practical implications for professionals working with families. The findings emphasize the importance of fostering a quality relationship, enabling families to better cope with stressors and maintain functioning amidst adversity. Strengthening family relationships, including cohesion, expressiveness, and conflict management, is equally vital. Strong family relationships can act as a buffer against stressors, potentially mitigating their detrimental effects on parental stress.
